# The Diatomite Grinding Technology Concept for the Protection of Diatomite Shells and the Control of Product Grading

**DOI:** 10.3390/ma17153662

**Published:** 2024-07-24

**Authors:** Agata Stempkowska, Tomasz Gawenda, Krzysztof Smoroń

**Affiliations:** 1 Faculty of Civil Engineering and Resource Management, Department of Environmental Engineering, AGH University, Mickiewicza 30 Av., 30-059 Kraków, Poland; gawenda@agh.edu.pl; 2 Specialized Mining Company GÓRTECH Sp. z o.o., 31-586 Kraków, Poland; k.smoron@diato.pl

**Keywords:** diatomite, mineral processing, technology conception

## Abstract

Diatomite deposits in Poland are located in the Podkarpackie Voivodeship, and the only active deposit is in Jawornik Ruski. Therefore, it is a unique material. Improved rock processing methods are constantly in demand. In the research presented here, we have used research methods such as X-ray diffraction (XRD), scanning electron microscope (SEM), particle shape analysis, and appropriate sets of crushing machines. Diatomite comminution tests were carried out on test stands in different crushers (jaw crusher, hammer crusher, high-pressure roller press, ball mill) using different elementary crushing force actions: crushing, abrasion, and impact, occurring separately or in combination. The machines were tested with selected variable parameters to obtain products with a wide range of grain sizes ranging from 0 to 10 mm. The ball mill (yield 87%, system C3) and the hammer crusher with HPGR (high-pressure grinding roller) (yield 79%, system D2 + D3) have the greatest impact on diatom shell release and accumulation in the finest 0–5 μm and 5–10 μm fractions. For commercial purposes, it is important to obtain very fine fractions while keeping the shells undisturbed.

## 1. Introduction

Diatomites are rocks that are formed from accumulated diatom shells as a result of the diagenesis process. These rocks are distinguished by specific properties and many applications. Diatomite often serves as a simple filler, but especially after modification, it can also be an active additive with many functions. Expensive chemical and physical functionalization methods have been developed to improve the properties of diatomite. Chemical modification of the diatomite surface through functionalization with coating reagents improves their ability to bind certain target compounds. This type of functionalization has been used to produce drug carriers, optical sensors, and adsorbents for water treatment, exchange, and filtration applications. Thermal functionalization of diatomites by calcination improves their physical properties, mainly compressive strength [1].

**Figure 1:**
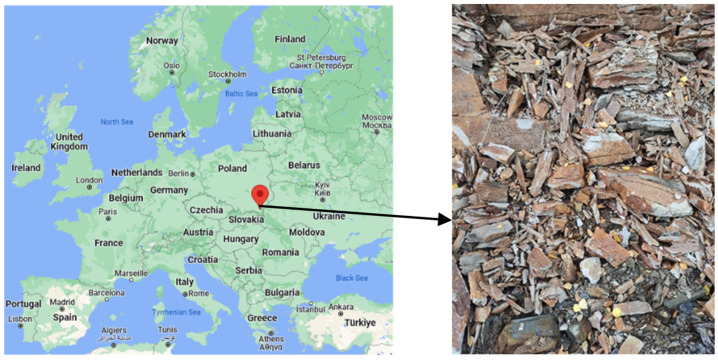
Location of diatomite deposits and an example image of the rock.

Diatomaceous earth deposits in Poland are found in the Podkarpackie province and the only active deposit is in Jawornik Ruski (Figure 1). In Poland, the extraction in 2021, according to data from the “Balance of mineral resources in Poland as of 31 December 2021”, oscillated approximately 0.98 thousand tons [2]. Pure diatomite is a highly sought after raw material. For many years, there has been a noticeable increase in interest in diatomite from many industries. On the world market, diatom raw materials are used in filtration and purification processes in the chemical and food industries (e.g., beer filtration). Insulating and soundproofing materials are often made from diatomite. A fairly common use for diatomaceous earth is as a sorbent for petroleum substances in various types of emergency spills (at petrol stations, airport runways, factory floors, water spills, etc.) as a pesticide carrier and in zoology as a bedding for pets. Pure diatomites are the most desirable, but those with a SiO_2_ content of more than 80% are not found in Poland. The use of diatomites is also quite common in industries such as construction, mechanical engineering (for removing coolants and lubricants), refractory materials, and the chemical industry. The great importance of diatomites lies in their use in agriculture, animal breeding, and broadly understood environmental protection as an effective material for the purification of water and sewage or immobilization of heavy metals in the soil [3,4]. Research by Aksakal et al. [4] showed that the use of diatomite significantly increases the stability of soil aggregates as well as the moisture and field capacity of sandy soil. The authors cited, such as Ye et al. [3], believe that the application of modified diatomite to contaminated soil increases the immobilization of toxic heavy metals and has soil potential to improve the microbiological activity. Synthetic zeolites and other sorbents can also be used for diatomites. Authors such as Grela et al., 2016, 2021, Łach et al., 2019, 2022, showed in their work that it is possible to produce complex zeolite structures from various types of raw materials that contain an increased content of SiO_2_ [5,6,7,8]. The possibility of obtaining Y-type zeolites from diatomites [9] and zeolites obtained by the microwave method [10] has been confirmed. Studies of diatomite with an increased clay mineral content have shown that heat treatment at 1000 °C causes the formation of mullite typical of this temperature. Diatomites contaminated with clay minerals, which are referred to as “diatomaceous clay”, have the potential as a basic raw material for the production of various ceramic materials, such as structural and thermal materials (various types of zeolites, water glass, amorphous SiO_2_, etc.) [11,12]. An interesting application of sintered diatomite is as orthopaedic magnesium implants [13]. Sintered diatomites can find applications as filters and membranes [14,15]. Appropriate calcination below the sintering temperature can improve the sorption performance of diatomite [16]. Diatomite can also be used as a carrier for phase change materials (PCM) [17,18,19]. The produced PCM composites have high thermal cycle efficiency and are used in medium-temperature thermal energy storage [20,21]. There are also attempts to use diatomite as an additive to concrete and cement binders [22,23,24]. Tests carried out with diatomite showed that the early strength of mortars using diatomite is lower than the strength of reference samples. However, after 28 days of seasoning, the strength increases, which may indicate the pozzolanic nature of this material. [25]. Many cement substitutes have a later setting time and the final strength is achieved only after 96 days [26]. In addition, higher resistance to freezing and thawing cycles of cement mortar with addition of diatomite was noted [27].

The great majority of scientific work focusses on learning about the properties of properly processed diatomites. There are relatively few studies on how to process the diatomite bedrock [28]. Some authors propose selective purification of diatomite through appropriate processing [29,30]. Therefore, the authors of this publication have conducted research and evaluation of the processing potential of this raw material. The authors have extensive experience in the grinding and purification of various types of raw materials [31,32,33,34] and especially in the innovative technology of producing shaped and unshaped aggregates for special applications [35]. Natural diatomite of good quality becomes rare and the demand is increasing. Intact diatom shells are the most valuable. Normal processing can damage the delicate shells. Depending on the physical properties of the material, i.e., its hardness, brittleness, strength, and susceptibility to comminution, the crushing parameters vary [36]. The crushing behavior of porous materials differs significantly from that of nonporous materials. Diatomites are rocks with specific porosity. Subsequently, concepts were developed for a diatomite grinding technology with a capacity of approximately 10–20 t/h, allowing the degree of granulation of the products to be controlled so that fractions with a wide range of grain sizes could be produced. The most important task was to minimize the destruction of the diatomite shells. In summary, the primary objective of the research described in this article was to develop a processing technology that can control the particle size distribution and quality of diatomite products.

## 2. Materials and Methods

### 2.1. Basic Physical Parameters, XRF Chemical Analysis, XRD Phase Composition Assessment, and Microscopic Images of the Feed

**Figure 2:**
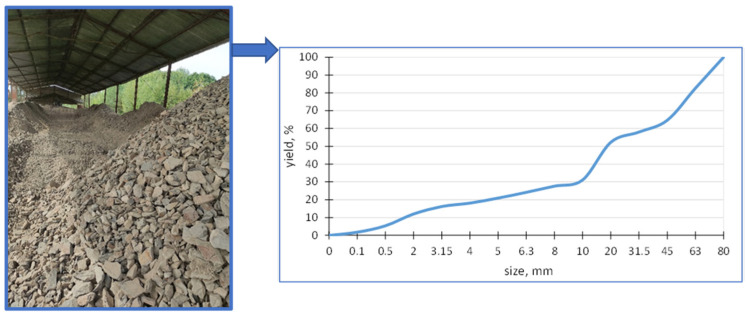
Diatomite feed delivered for testing and grain composition curve of diatomite feed.

The raw material supplied for testing from the Diatomite Mine in Jawornik Ruski, Figure 2, weighing about 400 kg, was characterized by a grain size from 0 to 80 mm. The Bond work index (Wi) in the ball mill was determined to be 10.1 kWh/Mg. The Bond work index is a measure of rock resistance to crushing and grinding and is determined using the Bond grindability test. Its value constitutes rock characteristics and is used for the design of industrial combustion plants [36]. It is a medium-hard raw material that can be easily crushed. Analysis of the composition of the grain size of the feed was carried out on an averaged sample according to PN-EN 933-1 [37] in sieves with a mesh size of 0.1; 0.5; 2; 3.15; 4; 5; 6.3; 8; 10; 20; 31.5; 45; 63; 80 mm. Figure 2 also shows the grain composition of the feed. The determination of moisture was carried out according to the PN-EN 1097-5 standard [38]. The moisture content of the test material batch was 10.03%.

The diatomites studied are rocks formed from the siliceous shells of unicellular algae called diatoms. Their shells are very durable and, after dying, sink to the bottom of water bodies where they form silica sediments. This process is accompanied by partial dissolution of the shells and clumping of the remaining ones with the released silica. The remaining cementing substance consists of clay minerals, chloritized muscovite, gypsum and iron compounds, and organic particles, which are impurities in the diatomite. To correctly assess the chemical composition of the material, an X-ray fluorescence (XRF) testing technique (Rigaku—Primini WDXRF spectrometer, Tokyo, Japan). Based on the averaged XRF analysis, the SiO_2_ content was 60.49 %wt., and the Al_2_O_3_ content was 21.86% by weight. In general, these values fall within the ranges presented by other researchers [17,39,40]. The complete elemental analysis is presented in Table 1.

Mineralogical studies using XRD X-ray analysis were carried out with a Panalytical model Empyrean instrument (Malvern, UK). The share of individual phases was determined using the Rietveld method. Measurements were made using monochromatic radiation with a wavelength corresponding to the Kα1 emission line in the angle range 5–90° on the 2 scale. Qualitative analysis of phase composition was carried out using the X‘Pert HighScore plus 3.0 Plus computer program developed by PANanalytical. The reference databases used were PDF-2 (2004) and the ICSD Database FIZ Karlsruhe (2012). Due to the characteristic structure of the diatomites, phase analysis was difficult. The main rock formation mineral is opal, which is an amorphous hydrated form of opal silica. It does not form crystals and is a solidified colloidal silica with a water content varying between 3–10 %wt. Noncrystalline forms are not recorded by X-ray diffraction (XRD) and become visible as a raised background. Table 2 shows the mineralogical composition of the samples before different treatment processes, which are described in detail later in this publication.

The table indicates that the diatomites consist mainly of more or less crystallized silica. The estimated content is in the range 25–56%. Such large discrepancies are due to the presence of opal-type silica, which has no ordered structure and gives disturbed spectral reflections. In other words, X-rays are not reflected from the plane of the crystal at a well-defined angle. In the diagram, the presence of such structures is observed as a raised background in the 2Ɵ 0–10 angle range [41,42]. The second phase giving strong reflections is muscovite and its derivatives observed at 50 %wt, and this is an overestimate value. Muscovite itself and the products of its alterations (chlorite minerals) are naturally present in diatomites, usually in amounts not exceeding several percent. Feldspars are also present in diatomites, usually in the form of albite in amounts up to a few percent. The high presence of silica suggests that the material will have abrasive properties on the working surfaces of grinding machines.

**Figure 3:**
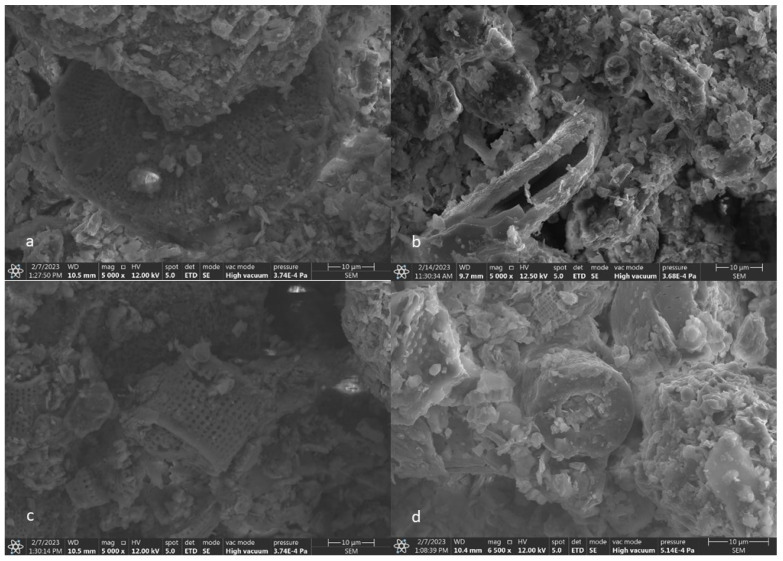
SEM images with diatoms of different types: (**a**) cylindrical, (**b**) disc shaped, (**c**) preserved both parts of the shell, and (**d**) filled with silica.

A study of the diatomite from the Jawornik Ruski mine, using SEM, electron microscopy, and EDS X-ray dispersive spectroscopy analysis (FEI, Nova NanoSEM 200) (Hillsboro, OR, USA), showed that the raw material consists of diatomite skeletons of various types, clay minerals (and derivatives), quartz, iron compounds (impurities), a small amount of feldspar and organic matter. The SEM images showed that the primary diatoms are predominantly disk shaped, plate shaped, and tube shaped, with the remaining minerals in the form of grains and spherical conglomerates, as shown in Figure 3.

**Figure 4:**
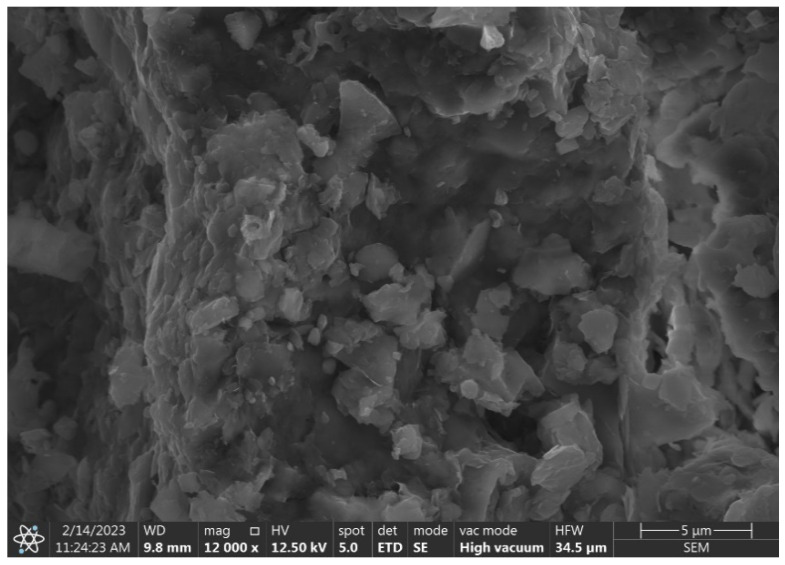
Silica recrystallized by geological processes from practically no preserved diatom shells.

**Figure 5:**
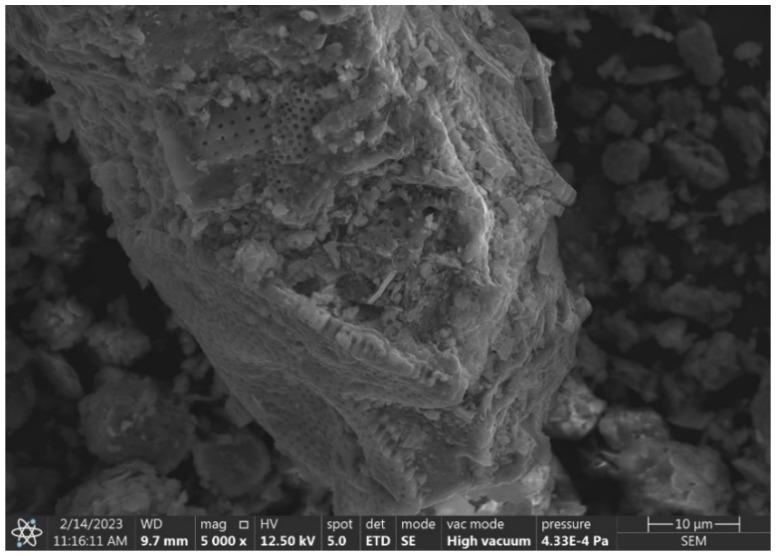
Conglomerate of diatom shells larger than 45 µm.

Disk-shaped diatoms present in the feed are approximately 50–60 μm in radius (Figure 3a,b), while cylindrical forms are much smaller in diameter up to 30 μm with varying elongation from 30 to 60 μm (Figure 3c). A large mass of silica is also observed, which comes from dissolved diatoms and recrystallization from the solution (Figure 3d, Figure 4 and Figure 5). Diatoms have a highly porous structure. Some diatoms form diagenetic conglomerates (Figure 5), which should be ground to release their sorption and parasite-fighting capacity. Therefore, the technological process of grinding should end with a deeper grinding in the mill to increase the impact on the grains. A large mass of silica is also observed, which originates from dissolved diatoms and recrystallization from solution (Figure 5). Such silica has the favorable property of fracturing to form sharp-edged forms. The proportion of diatoms and their coarser detritus (more or less damaged forms) was determined planimetrically and estimated at 20–60%, so the amount of armor is highly variable. The amount of silica binder varies accordingly, which was estimated to be between 30 and 80%.

**Figure 6:**
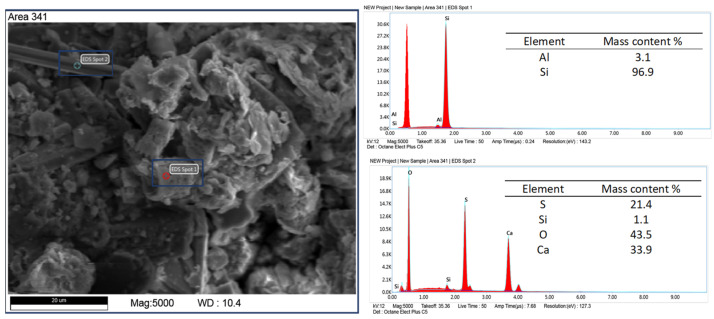
Analysis of a microarea diatom composed of silica (spot 1) and visible elongated gypsum crystals (spot 2).

**Figure 7:**
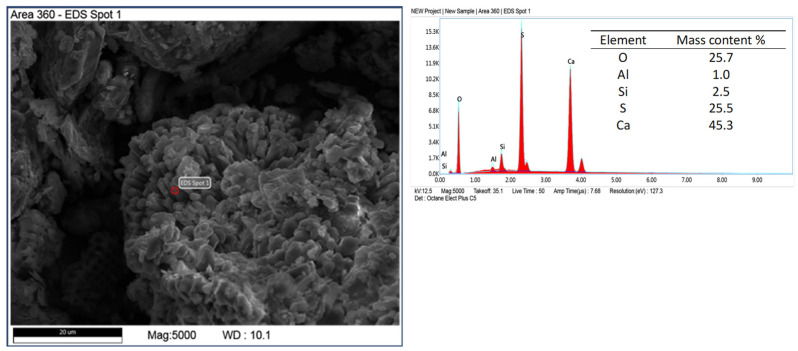
Gypsum brush form, feed sample.

**Figure 8:**
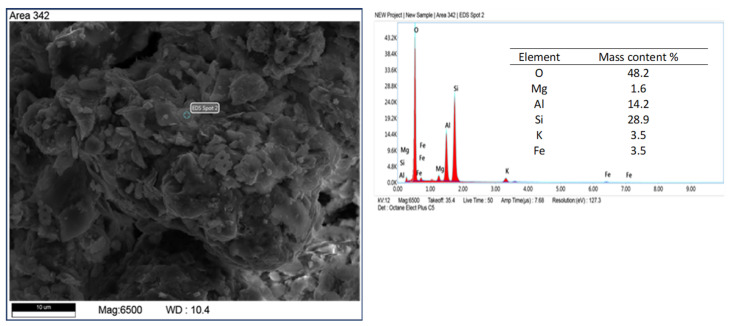
Other mineral forms present in diatomite, most likely chlorite.

**Figure 9:**
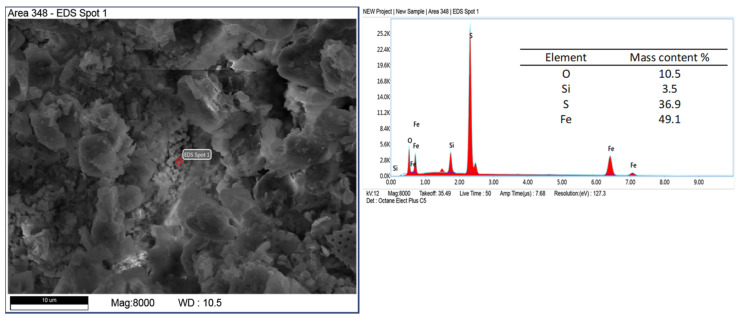
Iron-rich spherical forms identified as pyrite.

**Figure 10:**
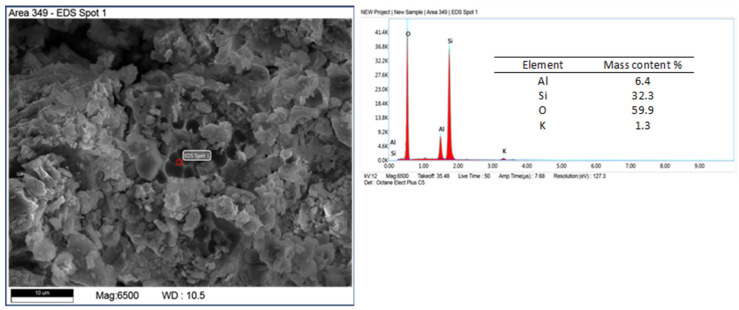
Silica flowstone form (opal).

**Figure 11:**
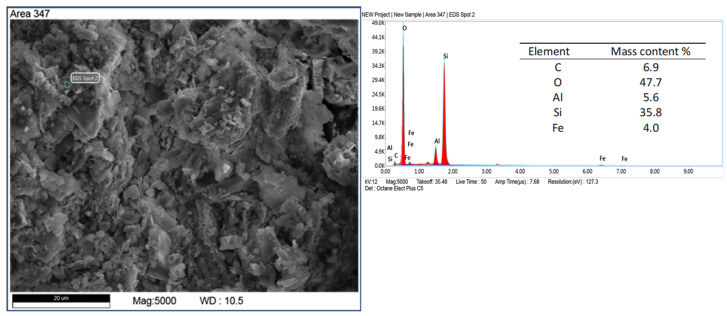
Presence of organic carbon.

Elemental analysis by X-ray dispersive spectroscopy (EDS) shows that the diatomite carapace is mainly composed of silica with minor amounts of alumina (Figure 6). This is a typical composition for diatomite rocks [43,44]. In addition to diatom shells and recrystallized silica, other mineral phases were observed in the samples in the form of elongated crystals, brush-like, flowstone-like, and spherical forms. Elemental analysis of the microareas on the surface of z-diatomite shows differences in the qualitative and quantitative chemical composition of these minerals. Figure 6, Figure 7, Figure 8, Figure 9 and Figure 10 show the differences in the chemical composition of the microareas. The elongated and brush forms are gypsum, as shown by the analysis in Figure 6 and Figure 7. Small amounts of organics were also detected—Figure 11.

Other mineral forms are in the form of crystals with more regular forms and isometric conformation. The chemical composition of these minerals is similar to that of mica (muscovite type) and transformed muscovite into chloritic (clinochlorite) forms (Figure 8). The occurrence of these minerals is confirmed by the XRD studies carried out. Spherical forms were also observed (Figure 9), which contain large amounts of iron in their chemical composition. Data from the literature indicate that this may be an impurity in the form of pyrite [45,46]. Iron in diatomites also occurs in the form of silicates, mainly clay minerals. Diatomites are largely composed of opal silica with a characteristic flowstone structure. It is largely amorphous, that is, it does not show an ordered internal structure. Such silica is observed in Figure 10. In addition to mineral forms, small amounts of organics are also present in diatomite—carbon content 6.9 %wt.—Figure 11.

### 2.2. Characteristics of the Grinding and Screening Machines Used in This Study

**Figure 12:**
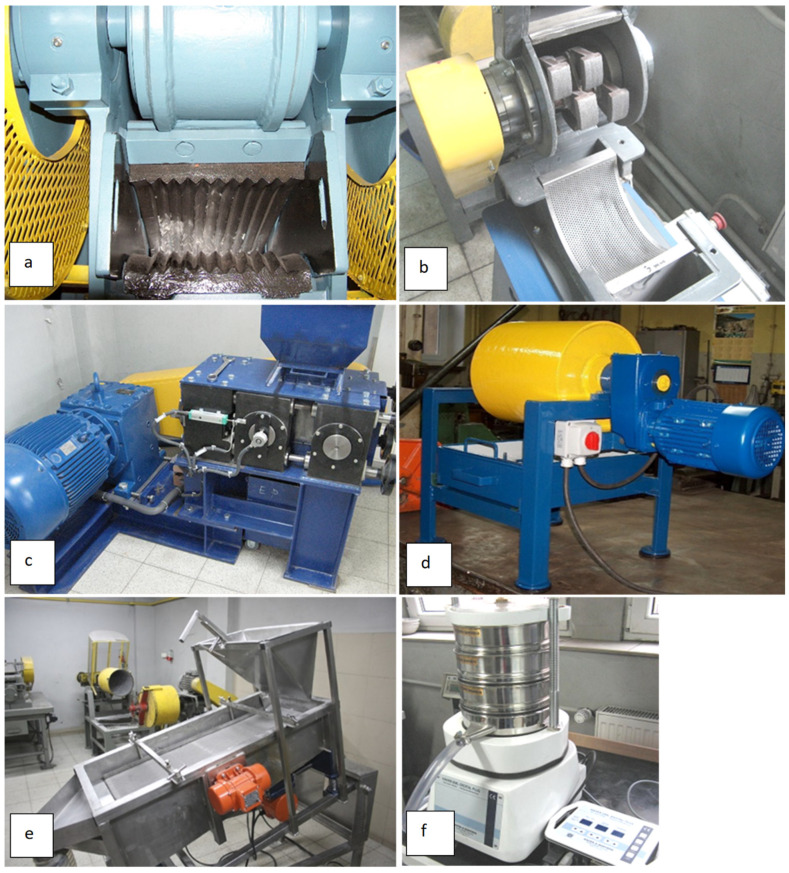
Crushing and screening machines: jaw crusher (**a**), hammer crusher (**b**), high-pressure grinding rolls (**c**), ball mill (**d**), vibrating screen (**e**), and shaker with analytical sieves (**f**).

Grinding and classification tests were carried out using test machine benches (Figure 12) located in the grinding and screening laboratory of the Department of Environmental Engineering of the AGH University of Science and Technology in Krakow. The test stand consists of a jaw crusher L44.41 from (Makrum, Bydgoszcz, Poland), a hammer crusher LKM 120 from Eko-Lab (Brzesko, Poland), a vibrating screen Eko-Lab (Brzesko, Poland), and a shaker with analytical sieves Haver EML Digital Plus (Hohenlimburg, Germany). The Bond ball mill and high-pressure grinding rolls were made at the Department of Environmental Engineering, AGH University of Science and Technology, Krakow, Poland.

**Figure 13:**
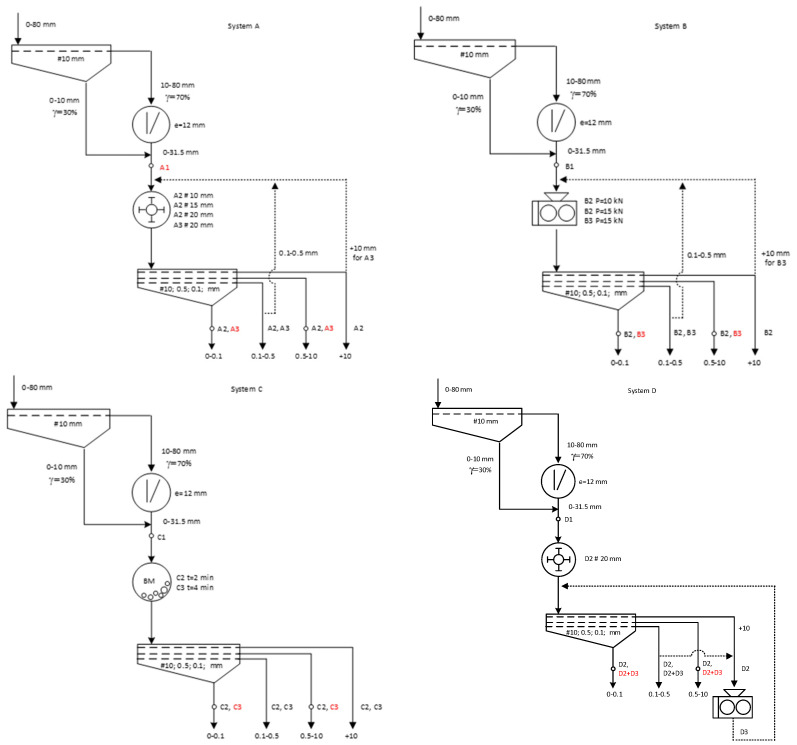

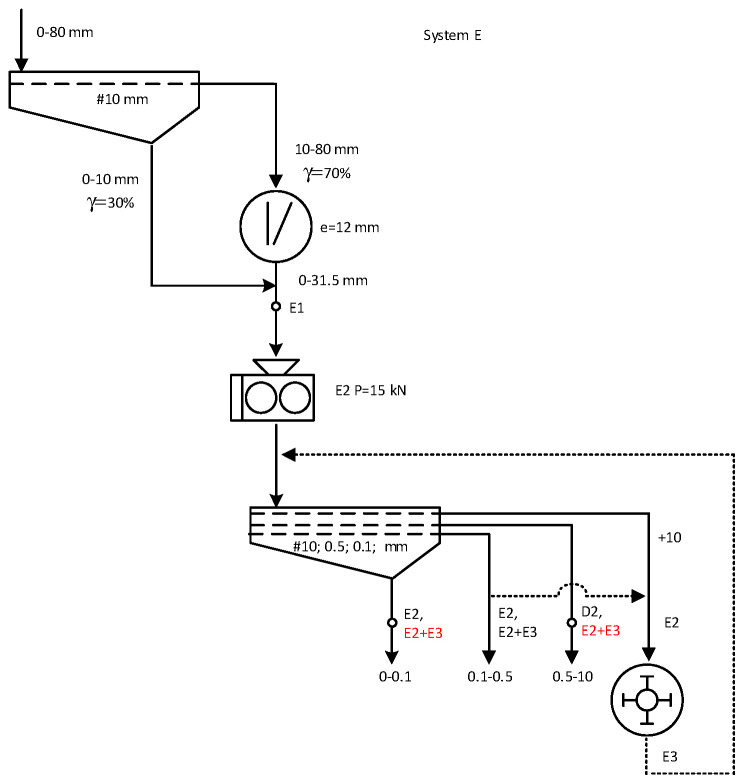
Diagrams of technological systems.

Five systems (Table 3) were developed with selected machines operating at specific technological parameters, with each technological system starting with precrushing in a jaw crusher in the first crushing stage, where the 0–31.5 mm product was then averaged as feed for crushing tests in subsequent machines. The systems were named as follows (Figure 13):

- System A with a jaw crusher and a hammer crusher;
- System B with a jaw crusher and a high-pressure grinding roll (HPGR);
- System C with a jaw crusher and a ball mill;
- System D with a jaw crusher, a hammer crusher and high-pressure grinding rolls (HPGR);
- System E with a jaw crusher, a high-pressure grinding rolls (HPGR) and a hammer crusher.

**Table 3:** Technological systems with crushers and their operating parameters.

Crusher, System	Operating Parameters
A1 #12 mm, jaw crusher, open circuit	outlet gap 12 mm
A2 #10 mm, hammer crusher, open circuit	grid 10 mm, rate 3000 1/min
A2 #15 mm, hammer crusher, open circuit	grid 15 mm, rate 3000 1/min
A2 #20 mm, hammer crusher, open circuit	grid 20 mm, rate 3000 1/min
A3 #20 mm, hammer crusher, closed circuit	grid 20 mm, rate 3000 1/min
B2 10 kN, HPGR, open circuit	roll pressure force, 10 kN, gap 3 mm, speed 0.5 m/s
B2 15 kN, HPGR, open circuit	roll pressure force, 15 kN, gap 3 mm, speed 0.5 m/s
B3 15 kN, HPGR, closed circuit	roll pressure force, 15 kN, gap 3 mm, speed 0.5 m/s
C2 2 min, ball mill, open circuit	milling time 2 min, Bond’s standards
C3 4 min, ball mill, open circuit	milling time 4 min, Bond’s standards
D2 + D3 #20 mm, hammer crusher + HPGR, closed circuit	grid 20 mm, roll pressure force, 15 kN
E2 + E3 #10 mm, HPGR + hammer crusher, closed circuit	roll pressure force, 15 kN, grid 10 mm

On the diagrams of technological systems (Figure 13), symbols have been marked with the letters A, B, C, D, and E, which denote the technological system associated with a particular comminution machine.

A number has been assigned to each letter along with the operating parameters of the machine on which the test was performed. For example, A1 denotes the product obtained in a jaw crusher operating at a discharge gap of 12 mm in the A system, while A3 #20 mm denotes the product obtained in a hammer crusher with a grate mesh size of 20 mm in a closed system. The red color in the figures indicates the collection of selected samples for various analyses, such as grain size and grain shape analyses according to Zingg classification. Furthermore, from these samples, comminution products of 0–0.1 mm were separated for evaluation of the diatomite phase composition by XRD, as well as for SEM analyses and evaluation of the destruction of diatomite armor destruction depending on the size of the fraction and the use of different comminution machines.

## 3. Results

Grinding tests were carried out on a feed jaw crusher, a hammer crusher, a high-pressure grinding roll (HPGR) and a ball mill at selected variable machine parameters. The products were subjected to grain size distribution, shape and SEM scanning microscopy analysis to assess the destruction of diatom shells.

### 3.1. Grinding Rates for All Systems

Table 4 summarizes the degrees of comminution (maximum and eighty per cent) for machines operating in different process systems and the flatness index FI for the 2–10 mm fraction [47]. The highest degrees of comminution were characterized by the HPGR roller press and the hammer crusher operating in the closed system with a crusher grate of 10 mm (value 4.4), followed by the closed system roller press (value 4.1). The highest content of unshaped grains FI is approximately 28–30%, which was for the hammer crusher operating in systems A2 and A3 and the lowest for the ball mill operating in systems C3 and C2.

Table 5 summarizes the outputs of the crushing products in the various grain classes for machines that operate in different process systems. Taking into account the possibility of obtaining outputs for the 0.5–10 mm fraction, it can be concluded that crushers operating in closed systems produce the highest shares, exceeding 83% (hammer crusher in the A3 closed system and a hammer crusher with HPGR in the D2 + D3 system). As the proportion of fines in the coarse fraction (0.5–10 mm) increases, the proportion of fines <0.1 mm decreases, ranging from 4 to 6% for closed systems. The open system with an A2 hammer crusher operating in a 10 mm grate is also notable, as almost 7% of the fine fraction <0.1 mm and almost 80% of the coarse fraction 0.5–10 mm can be obtained.

### 3.2. Analysis of Grain Shape in Comminution Products

Separate representative samples with grain sizes of 0–0.1 mm and 0.5–2 mm were submitted to KAMIKA Instruments (Warsaw, Poland) for grain shape analysis according to the Zingg classification. This study used an optical electronic simulation of grain particle measurements according to mechanical sieves in the AWK 3D instrument (Warsaw, Poland). The basic method of classifying three-dimensional particles is Zingg characterization [48]. Generally, this is the analysis of the shape of particles which can be divided into 4 categories: shape, sphericity, flatness, and roundness. It is based on calculating the ratio between the three measured dimensions. The dimensions of a given particle are arranged from the smallest to the largest and denoted as follows:

- The smallest dimension,
- Intermediate dimension, and
- The largest dimension.

Then, two ratios are calculated: the ratio of the smallest to the average dimension a/b and the ratio of the intermediate to the longest dimension b/c. Depending on these ratios, particles are classified as spheres, discs, cylinders, and wedges (plates).

Table 6 summarizes the results of the samples analyzed in terms of shape in the grain size range of 0–0.1 mm, showing that system B3 with a roller press had the lowest spherical grain content (almost 87%) and system E2 + E3 with a roller press and a hammer crusher had the highest spherical grain content of spherical grains (91.5%). This slight variation in the shape of the grains in the finest 0–0.1 mm products may be due to the fact that the type of crushing machine and, more specifically, its elementary crushing forces from the working elements do not have as great an effect on the finest grains as in the case of coarser fractions. The formation of fine grains (dust) may be due to the effect of the coarser grains grinding into each other. However, a deeper milling of the raw material in the third grinding stage will be important, as confirmed by the analysis of the shape of the grain performed in the 0–10 µm range (Table 7). From the results presented, it can be seen that the proportion of spherical grains in the fine 0–10 µm expression range 0–10 m decreases from approximately 90% to approximately 50% (the exception is the roller press system with the E2 + E3 hammer crusher, since the proportion of spherical grains is 71%). As the proportion of spherical grains decreases, the content of flat grains (disc and lamellar) increases for the machines tested and is at a level of approximately 29 to 49%. The grain shapes for the coarser 0.5–2 mm fraction (Table 8.) vary considerably. The smallest number of spherical grains (almost 50%) was obtained for the ball mill in the C3 layout, with approximately 22% of flat grains (lamellar and disc). If one were to consider the layout and machines that produce the most regular grains (spherical and cylindrical), it would be the B3 layout with a roller press (85%) and the least regular grains would be the D2 + D3 layout with a hammer crusher and roller press (71%). So the same D2 + D3 system produces the most flat grains (lamellar and disc) at 29%.

### 3.3. SEM and EDS Microscopic and Chemical Analysis of Grinding Products and Evaluation of Diatom Shell Destruction

**Figure 14:**
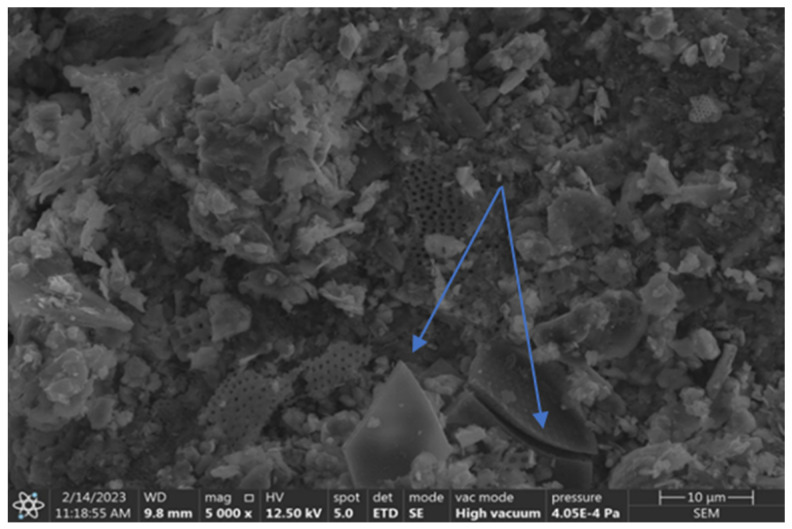
Visible sharp-edged silica and crushed diatom shells.

**Figure 15:**
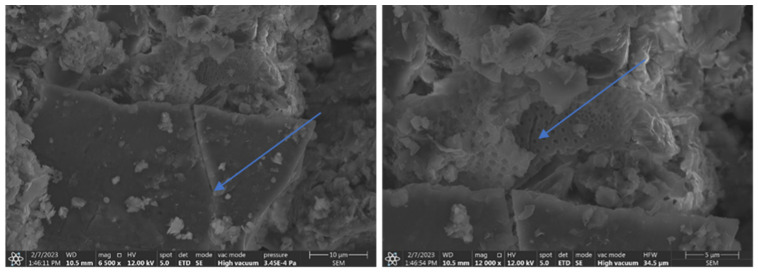
Visible incomplete crushing after pressure pressing—visible microcracks on silica crystal (**left**) and diatom shell (**right**).

A further scanning microscopy study was carried out after various rock crushing processes. In Figure 14, fractured sharp edged silica can be observed, sample D2 + D3 (hammer crusher and HPGR), next to regular diatom shells with spiked edges. The effect of hammer crusher grinding will benefit parasite control. The development of recrystallization-derived silica surfaces and amorphous opal forms results in increased wettability to polar liquids [49]. Wetting of solids by liquids is the result of interactions between the liquid and the solid phase. A liquid wets a solid when the interactions between the molecules in the solid and the liquid (adhesion) are stronger than those between the molecules in the liquid (cohesion). The surface of silica, due to supersaturation with oxygen, becomes negatively charged, causing the attraction of water dipoles [50]. Thus, parasite-inhaled diatomites have a drying and destructive effect on them. Furthermore, such a property may also be important for the adsorption of heavy metal cations. However, this is a complex process that depends on many factors including temperature, pH, concentration, or contact time of the substance. When a pressure roller press was used, the phenomenon of incomplete crushing of the grains was observed. This manifests itself in the form of microcracks (Figure 15). This phenomenon can be interpreted as favorable in terms of the formation of new sharp edges on the grains in the case of parasite control and unfavorable in the case of sorbent production due to poor strength and excessive subgrain formation during material packing and transport.

**Figure 16:**
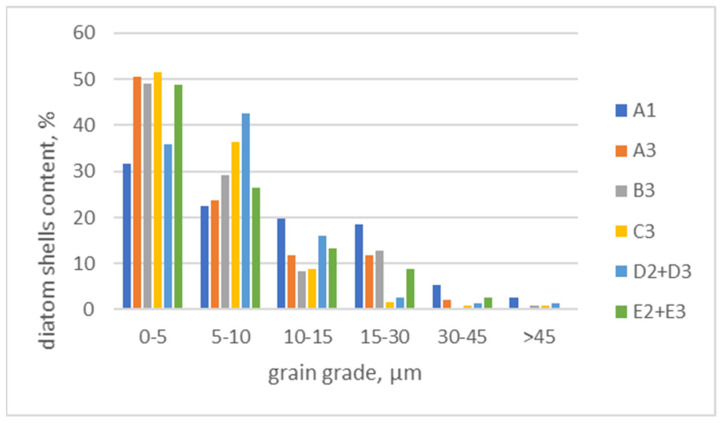
Quantitative analysis of diatom shells in relevant grain classes after different grinding processes.

Microscopic images of the samples after various grinding processes were also analyzed. The number of well-preserved diatom shells was counted, assigning them to the appropriate grain class as shown in the graph (Figure 16). As the grain size of the diatomite decreases, the content of well-preserved diatomite shells increases. This can be explained by their release from the remaining rock formation minerals and their accumulation in the finest fractions (transition from coarser to finer classes) as a result of the rock fragmentation process. This principle is confirmed by the smallest proportion of diatom shells in the classes 0–5 μm and 5–10 μm classes obtained in the jaw crusher after the first crushing stage (coarse crushing) at a total of 55%. The ball mill (87%) (system C3) and the hammer crusher with HPGR (79%) (system D2 + D3) have the greatest influence on the release of diatom shells and their accumulation in the finest fractions of 0–5 µm and 5–10 µm. The Zingg grain shape analysis shows a clear increase in the proportion of flat grains (disc and plate grains) in the range 0–10 µm range compared to the fraction 0–100 µm fraction. Therefore, the use of hammer crushers for the first (primary) and second (secondary) comminution stages and a ball mill for the third comminution stage (final milling) is recommended for the construction of the technological system for the comminution of diatomites. Second and third-stage grinding should be performed on the dried raw material.

### 3.4. The Concept of the Technological System

**Figure 17:**
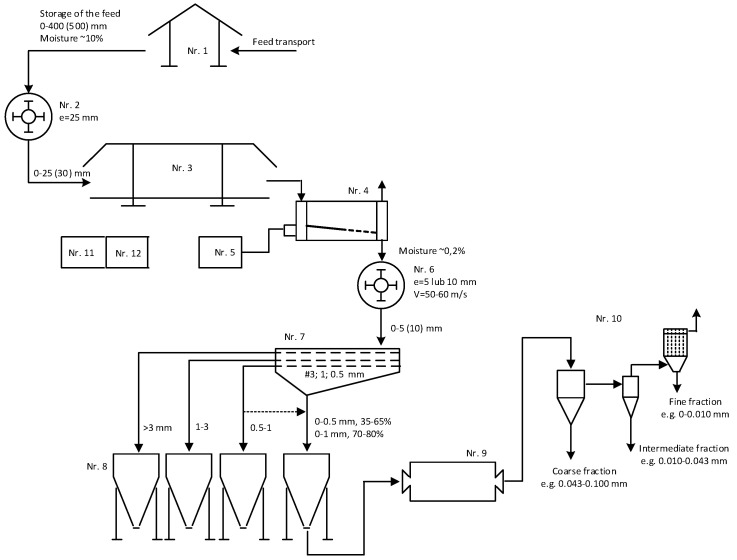
Schematic design of a technological system for grinding and classification with drying of diatomite with a capacity of up to 20 Mg/h.

On the basis of diatomite grinding tests carried out in different grinding machines and SEM microscopic analysis, the destruction of the diatomite carapace was assessed as a function of fraction size and the impact of using different grinding machines. The greatest impact on the release of diatom shells and their accumulation in the finest fractions of 0–5 μm and 5–10 μm fractions is exerted by the ball mill (87%). Figure 17 shows a schematic diagram of the concept of the technological system for dual-stage grinding and classification with diatomite drying to produce sand, grit and silt fractions in different grain size ranges depending on market needs. The planned maximum capacity of the system is 20 Mg/h. The feedstock has a maximum grain size of up to 500 mm, with 80% of the grains smaller than 350 mm. The moisture content of the feedstock is approximately 10% and the assumed moisture content of the products is approximately 0.2%.

The technological circuit, presented in Figure 17, consists of 12 facilities:

1. Stone storage and transport facility for the crushing plant.

Feedstock should be pre-stored in front of the shredding facility to ensure continuous production and pre-dried. The moisture content of the raw material is approximately 10% and can increase during certain periods of weather, so storage under a roofed shelter is recommended to stabilize it. Conveyor transport with scales and a metal separator will ensure safe, continuous, and controlled feed delivery to the crusher.

2. Crushing facility with a hammer crusher with a 25 mm grid.

The primary impact crusher should grind the raw material to at least a grain size of less than 25 mm. Materials with a finer grain size will be dried more effectively in the pre-drying facility (3) and in the rotary dryer (4) and will have a more favorable effect on the second stage crushing process (6), thus reducing the energy consumption of the process.

3. Raw material pre-drying facility with roofing.

It is recommended that the drying medium be used and distributed in the air ducts of the facility.

4. Raw material drying facility in a rotary dryer with loading system.

The rotary drum dryer is responsible for the important process of drying the raw material to a moisture content of less than 0.2%. The dryer should be integrated with a loading hopper into which the raw material will be fed by a loader with a vibrating feeder with the possibility of capacity regulation and a belt conveyor feeding the feedstock to the dryer.

5. A fuel oil or gas tank facility with a heat burner in the drying system.

Hot gases, a mixture of air and fuel combustion products, will be used to dry the material in the drum dryer, which will be fed from the tank station.

6. Crushing facility with a hammer crusher, 5 or 10 mm grate (interchangeable), and variable linear speed 50–60 m/s.

The hammer crusher should have the ability to change grates and vary the linear speed of the rotor smoothly to adapt the size of the product granulation to the requirements of the customers. It should be connected to a screen grader facility with a dust collection system.

7. Screen classification facility with multideck staggered motion screen with production dust collection system.

This is the first stage of classification in the sieve shaker for separating sand and grit fractions in different grain size ranges according to market needs. It is also possible to combine the finest fractions to 0–1 mm in the screen in the event of an increase in the demand for fine flour products below 0.1 mm and to direct them to be milled in the ball mill at Facility 9 and then to be directed to the second stage of pneumatic classification.

8. Silos collection and product packaging facility.

At least 4 silos are provided for the storage of different fractions of finished diatomite products in different fractions.

9. Ball mill grinding facility with dust extraction system.

A ball mill for homogenizing fine flour into dusty fractions and activating their surface and increasing the proportion of unformed grains by crushing and abrasion. Integrated with a pneumatic classification system in the separators. The versatility and ease of adaptation of its operation will not limit the grinding of coarser fractions if there is a need to increase the proportion of dusty fraction production. In the case of secondary agglomeration of the milled product, it is possible to use surfactants to facilitate milling and classification.

10. Pneumatic classification facility in an open-circuit air cyclone separator with dust extraction system in the filters.

This is the second stage of classifying dust fractions, where the coarse, intermediate and fine dust fractions are separated and adapted to market needs by grain size range. The main task of the cyclone is to relieve the filter. Depending on the cyclone’s performance, it is possible to achieve certain differences in the grain size of the product received from the cyclone and the fabric filter. The facility should include bagging stations with a dosing and weighing system.

11. Energy facility—power, supply, control.

The facility should be equipped with the necessary power and control in all objects of measurement and automation operations, which will occur under the supervision of a programmed control or visualization system.

12. Land development.

It is necessary to locate facilities not directly related to production, but in compliance with environmental, sanitary and social issues, health and safety requirements, and fire protection, as defined by the relevant regulations.

The choice of a hammer crusher in the two crushing stages is dictated by the fact that hammer crushers are used to crush medium-hard raw materials and wet materials up to several percent. They can achieve a relatively high degree of crushing compared to other crushers on the market. In addition, the predominance of the main comminution action, the impactor, entails a high selectivity of the comminution, which will affect the release of well-preserved diatom shells from the remaining rock-forming minerals and the accumulation of the finest fractions. The proposed crushing method, characterized by a two-stage crushing process and the possibility of freely and very widely selecting the linear speed of the crushing elements and varying the size of the grate outlet gap, allows a full and more effective mechanical activation of the manufactured product. To obtain the finest dust fractions, a ball mill should be used at the tertiary grinding stage, which will have a beneficial effect on the shape of the grains and the release of diatom shells, as confirmed by studies. With a ball mill, it is possible to control its operating parameters to a large extent by changing the size and weight of the grinding medium, grinding time, feed weight and grain size of the feed. This technological concept allows us to obtain the best possible quality of diatomite products from the tested materials.

## 4. Summary and Conclusions

- The highest comminution ratios were characterized by the roller press and the hammer crusher operating in the closed system with a crusher grate of 10 mm. It can be concluded that the crushers operating in closed systems produce the output of more than 83%, these are: a hammer crusher in the A3 closed system and a hammer crusher with HPGR in the D2 + D3 system.
- The yields of fractions below 0.01 mm in crushers are low, with the highest output of 0.25% achievable in a C3 system with a ball mill. The ball mill shows great potential, and the system should be terminated at the third crushing stage. It has a dominant function for the finer feed and should work in an open system with the dried material. In addition, it will have a beneficial effect on the shape of the grains and the release of diatom shells, which has been confirmed by studies presented in this article. It should be noted that the damp material fed into the mill will cause the raw material to cling to the roller and grinders.
- Crushing with high-pressure grinding rolls will be problematic. The grains obtained after HPGR are weak and tend to self-crumb. This is a feature of the product caused by acquired microcracking of grains as a result of high-pressure forces between the rollers in the press chamber on the material being shredded. This was visualized in photos taken in SEM.
- The shape analysis according to the Zingg classification in the grain size range of 0–0.1 mm shows that system B3 with a roller press had the lowest spherical grain production (almost 87%) and system E2 + E3 with an HPGR and a hammer crusher had the highest spherical grain production of spherical grains (91.5%).
- Samples of crushed materials were analyzed under an electron microscope in different crushers and machine systems. It was observed that, as the grain size of the diatomite decreased, the content of well-preserved diatomite carapaces increased. This can be explained by their release from other rock-forming minerals and their accumulation in the finest fractions as a result of the crushing process.
- A conceptual scheme of a technological system for dual stage grinding and classification together with diatomite drying, was proposed for the production of sand, grit, and silt fractions in various grain size ranges with a capacity of up to 20 mg/h. The technological system consists of 12 facilities.
- Appropriate processing of diatomite increases its potential for use in filtration, adsorption, and concrete additives and eliminates the challenges and limitations that can be encountered in these applications.

## Figures and Tables

**Table 1:** Averaged oxide composition of diatomite obtained by the XRF method.

Component	Concentration [%wt.]
Na_2_O	1.19
MgO	1.44
Al_2_O_3_	21.86
SiO_2_	60.49
P_2_O_5_	0.47
SO_2_	1.37
K_2_O	6.25
CaO	1.47
TiO_2_	1.10
Cr_2_O_3_	0.09
MnO_2_	0.21
Fe_2_O_3_	3.80
CuO	0.04
ZnO	0.04
Rb_2_O	0.07
SrO	0.03
ZrO_2_	0.07

**Table 2:** Phase composition of a diatomite sample.

Identified Mineral Phase	Estimated Amount of Mineral Phase in the Sample [%wt.]					
	A1, A2	A3	B2, B3	C2, C3	D2, D3	E2, E3
Quartz	35	27	38	56	29	25
Muscovite	51	50	50	25	59	57
Clinochlore	4	6	2	4	3	4
Orthoclase	5	11	-	6	3	4
Albite	5	6	11	11	9	10

**Table 4:** Degrees of comminution for different machines working in different systems and flakiness index for fractions 2–10 mm.

Crusher, System	S_max_	S_80_	FI [%]
A1 #12 mm, jaw crusher, open circuit	2.5	3.3	28.5
A2 #10 mm, hammer crusher, open circuit	3.2	3.8	29.8
A2 #15 mm, hammer crusher, open circuit	2.1	2.6	29.1
A2 #20 mm, hammer crusher, open circuit	1.6	2.3	27.9
A3 #20 mm, hammer crusher, closed circuit	3.2	2.9	29.3
B2 10 kN, HPGR, open circuit	1.0	1.5	16.8
B2 15 kN, HPGR, open circuit	1.0	1.4	17.1
B3 15 kN, HPGR, closed circuit	3.2	4.1	23.3
C2 2 min, ball mill, open circuit	1.0	1.2	11.2
C3 4 min, ball mill, open circuit	1.0	1.5	7.2
D2 + D3 #20 mm, hammer crusher + HPGR, closed circuit	3.2	3.7	22.4
E2 + E3 #10 mm, HPGR + hammer crusher, closed circuit	3.2	4.4	23.8

**Table 5:** Product outputs in different grain classes for different machines in different systems.

Crusher, System	γ_0–0.01_ [%]	γ_0–0.043_ [%]	γ_0–0.1_ [%]	γ_0.5–10_ [%]
A1 #12 mm, jaw crusher, open circuit	0.04	1.1	2.6	48.9
A2 #10 mm, hammer crusher, open circuit	-	-	6.8	79.3
A2 #15 mm, hammer crusher, open circuit	-	-	4.1	82.2
A2 #20 mm, hammer crusher, open circuit	-	-	4.2	73.6
A3 #20 mm, hammer crusher, closed circuit	0.08	2.4	5.0	83.2
B2 10 kN, HPGR, open circuit	-	-	3.9	63.5
B2 15 kN, HPGR, open circuit	-	-	3.4	64.9
B3 15 kN, HPGR, closed circuit	0.12	3.0	5.9	80.4
C2 2 min, ball mill, open circuit	-	-	8.0	24.5
C3 4 min, ball mill, open circuit	0.25	4.8	8.5	31.5
D2 + D3 #20 mm, hammer crusher + HPGR, closed circuit	0.09	2.2	4.6	83.2
E2 + E3 #10 mm, HPGR + hammer crusher, closed circuit	0.05	2.4	5.4	79.0

**Table 6:** Percentage distribution of grain shapes according to Zingg classification, test grain size range 0–0.1 mm.

Crusher, System	Spheres	Cylinders	Plates	Discs
A1 #12 mm, jaw crusher, open circuit	90.0	0.0	6.0	4.0
A3 #20 mm, hammer crusher, closed circuit	88.0	0.0	6.8	5.2
B3 15 kN, HPGR, closed circuit	86.8	0.0	7.7	5.6
C3 4 min, ball mill, open circuit	90.1	0.0	5.1	4.8
D2 + D3 #20 mm, hammer crusher + HPGR, closed circuit	89.5	0.0	5.7	4.9
E2 + E3 #10 mm, HPGR + hammer crusher, closed circuit	91.5	0.0	4.0	4.5

**Table 7:** Percentage distribution of grain shapes according to Zingg classification, test grain size range 0–0.01 mm.

Crusher, System	Spheres	Cylinders	Plates	Discs
A1 #12 mm, jaw crusher, open circuit	52.5	0.0	26.0	21.6
A3 #20 mm, hammer crusher, closed circuit	51.0	0.0	21.1	27.9
B3 15 kN, HPGR, closed circuit	51.7	0.0	27.3	21.0
C3 4 min, ball mill, open circuit	54.0	0.0	20.4	25.7
D2 + D3 #20 mm, hammer crusher + HPGR, closed circuit	52.5	0.0	24.3	23.2
E2 + E3 #10 mm, HPGR + hammer crusher, closed circuit	71.1	0.0	0.8	28.0

**Table 8:** Percentage distribution of grain shapes according to Zingg classification, test grain size range 0.5–2.0 mm.

Crusher, System	Spheres	Cylinders	Plates	Discs
A1 #12 mm, jaw crusher, open circuit	58.0	22.2	4.6	15.2
A3 #20 mm, hammer crusher, closed circuit	56.0	21.0	8.1	14.9
B3 15 kN, HPGR, closed circuit	62.8	22.1	2.9	12.3
C3 4 min, ball mill, open circuit	49.6	28.2	7.9	14.3
D2 + D3 #20 mm, hammer crusher + HPGR, closed circuit	51.2	19.9	9.0	19.9
E2 + E3 #10 mm, HPGR + hammer crusher, closed circuit	61.0	20.5	4.3	14.2

## Data Availability

No data was used for the research described in this article.
